# Silicone Breast Implant as a Hidden Cause of Fever: A Diagnostic Pitfall

**DOI:** 10.7759/cureus.89083

**Published:** 2025-07-30

**Authors:** Kotaro Kunitomo, Fumitaka Yoshimura, Junko Kubosaki, Takahiro Tsuji

**Affiliations:** 1 General Medicine, National Hospital Organization Kumamoto Medical Center, Kumamoto, JPN

**Keywords:** autoimmune/inflammatory syndrome induced by adjuvants (asia), fever of unknown, mri, potential pitfall for misdiagnosis, silicone breast implant

## Abstract

A 56-year-old woman developed a persistent fever following right transurethral ureterolithotripsy. Approximately two weeks prior to the onset of fever, she had sustained chest trauma and had a 20-year history of bilateral silicone breast implants. Physical examination, including breast assessment, was unremarkable. Blood cultures and CT revealed no identifiable source of infection. Despite treatment with broad-spectrum antibiotics and ureteral stenting, her fever and elevated CRP levels persisted. MRI revealed irregular margins and internal high-signal linear structures in the left breast implant on T2-weighted imaging, suggestive of intracapsular rupture. Autoimmune/inflammatory syndrome induced by adjuvants (ASIA) was suspected, and bilateral implant removal was subsequently performed. Her symptoms resolved following surgery. The diagnosis of ASIA was supported by fulfillment of three major criteria: exposure to silicone, presence of typical systemic symptoms (fever, fatigue, and sleep disturbance), and resolution of symptoms after implant removal. ASIA is a rare immune-mediated condition triggered by exposure to adjuvants such as silicone breast implants. Diagnosis can be especially challenging in the absence of local symptoms at the implant site. This case highlights the importance of considering ASIA in patients presenting with unexplained systemic inflammation and a history of silicone implants, even when local signs are lacking. MRI played a key role in detecting the implant rupture and guiding appropriate intervention.

## Introduction

Adjuvants are substances designed to enhance the effects of another concurrently administered agent [1]. They primarily exert their modulatory effects by stimulating the immune system and are commonly found in medical and therapeutic products such as vaccines, silicone breast implants, mineral oils, and cosmetics. Although generally considered safe, adjuvants can, in rare instances, trigger autoimmune responses. Autoimmune/inflammatory syndrome induced by adjuvants (ASIA) is an immune-mediated condition that arises following exposure to these substances [1].

Silicone, in particular, has been shown to induce autoimmunity through immune activation and molecular mimicry [1]. Silicone implants can lead to acute inflammation by increasing cytokine levels, resulting in the formation of fibrous capsules around the implants. These capsules typically contain CD4+ lymphocytes, macrophages, and giant cells, forming silicone granulomas. Additionally, cross-reactivity with glycosaminoglycans in connective tissues has been reported.

Clinically, ASIA presents with a wide spectrum of nonspecific systemic symptoms, including chronic fatigue, arthralgia, low-grade fever, myalgia, and neurocognitive disturbances. In cases related to silicone breast implants, ASIA often includes local symptoms such as breast pain, swelling, or redness, which commonly prompt imaging investigations. However, in the absence of overt local signs, the diagnosis can be easily overlooked, particularly in patients presenting with persistent fever [1-3]. This poses a diagnostic challenge for general physicians and hospitalists who may encounter patients with unexplained systemic inflammation.

This case report highlights an atypical presentation of ASIA, marked by the absence of breast-related symptoms. In this case, breast MRI was instrumental in identifying implant rupture and guiding appropriate clinical intervention.

## Case presentation

A 56-year-old woman developed a persistent fever following right transurethral ureterolithotripsy. Suspecting a urinary tract infection, clinicians placed a ureteral stent and initiated empirical broad-spectrum antibiotics - meropenem, vancomycin, and clindamycin - for approximately three weeks. However, her fever persisted. Two weeks prior to symptom onset, she had sustained blunt trauma to the left side of her face and chest after a fall. Her medical history included bilateral silicone implants placed 20 years earlier, a brain abscess 10 years ago, hypertension, and dyslipidemia. She also reported experiencing fatigue and sleep disturbances.

At presentation, her temperature was 37.0°C, and other vital signs were within normal limits. Physical examination revealed no tenderness, swelling, redness, or any localized findings in the breast region. Table 1 shows the laboratory test results. CRP was elevated at 9.8 mg/dL. Aspartate aminotransferase, alanine aminotransferase, and gamma-glutamyl transpeptidase levels were mildly elevated. Antinuclear antibody testing was negative.

**Table 1:** Laboratory test results with corresponding reference ranges

Parameter	Result (unit)	Reference range (unit)
White blood cell count	5.56 × 10³/μL	3.3-8.6 × 10³/μL
Hemoglobin	10.7 g/dL	11.6-14.8 g/dL
Platelets	33.9 × 10⁴/μL	15.8-34.8 × 10⁴/μL
Aspartate aminotransferase	67 U/L	13-30 U/L
Alanine aminotransferase	66 U/L	7-23 U/L
Gamma-glutamyl transpeptidase	104 U/L	9-32 U/L
Alkaline phosphatase	105 U/L	38-113 U/L
Blood urea nitrogen	13.0 mg/dL	8.0-20.0 mg/dL
Creatinine	0.72 mg/dL	0.46-0.79 mg/dL
Creatine kinase	331 U/L	59-248 U/L
Sodium	139 mEq/L	138-145 mEq/L
Potassium	3.9 mEq/L	3.6-4.8 mEq/L
Chloride	100 mEq/L	101-108 mEq/L
Free T4	1.2 ng/dL	0.9-1.7 ng/dL
Thyroid-stimulating hormone	1.50 μIU/mL	0.61-4.23 μIU/mL
Antinuclear antibody	<40	<40
CRP	9.80 mg/dL	<0.14 mg/dL

**Figure 1:**
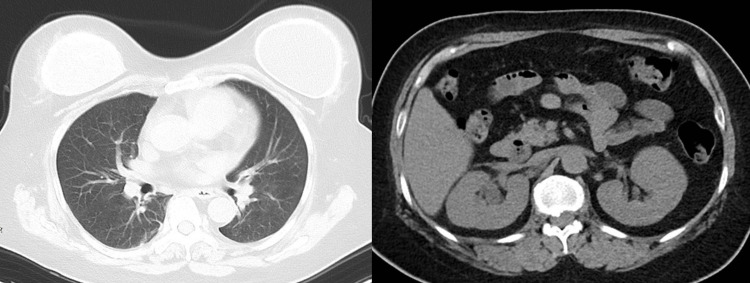
CT images obtained at admission Chest CT (left) and abdominal CT (right) showed no apparent abnormalities.

Blood and urine cultures obtained prior to the initiation of antibiotic therapy were negative. CT revealed no identifiable source of infection (Figure 1).

**Figure 2:**
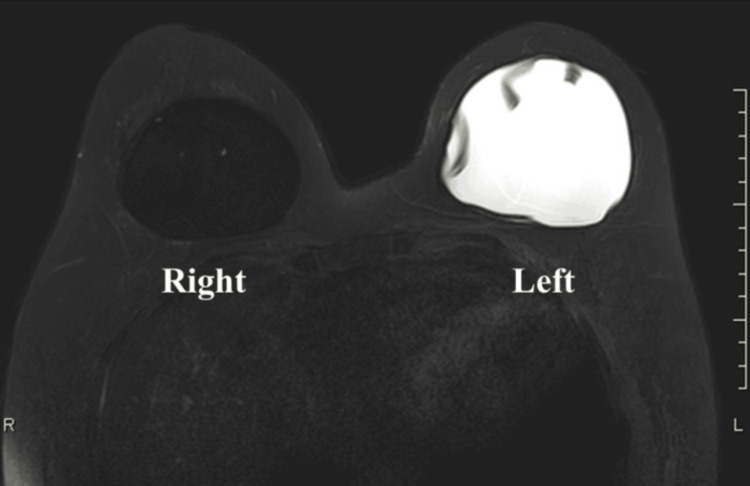
T2-weighted MRI of the breasts The left silicone implant shows irregular margins and internal linear high-signal structures on T2-weighted imaging, suggestive of intracapsular rupture. The right breast appears normal.

Although the patient had no local symptoms in the breast, breast MRI was performed due to her long history of silicone implants, persistent fever, and recent chest trauma prior to symptom onset. MRI revealed irregular margins and internal linear high-signal structures on T2-weighted imaging in the left implant, suggestive of intracapsular rupture (Figure 2). The right breast appeared normal.

Based on the MRI findings, we suspected that rupture of the left implant had triggered ASIA. The diagnostic criteria for ASIA are presented in Table 2 [1].

**Table 2:** Diagnostic criteria for ASIA Diagnosis is established by meeting either two or more major criteria or one major criterion plus two or more minor criteria. ASIA, autoimmune/inflammatory syndrome induced by adjuvants

Major criteria	Minor criteria
1. Exposure to external stimuli (infection, vaccine, silicone, or adjuvant) before the onset of symptoms	1. Appearance of antibodies directed against the suspected adjuvant
2. Appearance of typical clinical manifestations:	2. Secondary clinical manifestations (e.g., irritable bowel syndrome and interstitial cystitis)
a. Myalgia, myositis, or muscle weakness	3. Development of an autoimmune disease
b. Arthralgia and/or arthritis	4. Presence of antigens specific for human leukocytes
c. Chronic fatigue, unrefreshing sleep, or sleep disturbances	
d. Neurological manifestations	
e. Cognitive impairment or memory loss	
f. Fever	
3. Typical histological findings after biopsy of the affected organs	
4. Improvement of symptoms following removal of the offending agent	

With informed consent, bilateral implant removal was performed. Following removal of the silicone implants, the patient’s body temperature returned to the normal range, and her CRP levels gradually decreased over time. No recurrence of symptoms was observed. Histopathological examination of the pericapsular tissue surrounding the silicone breast implants revealed fibrous connective tissue with calcification and hyalinization. Although these findings were nonspecific, they were not inconsistent with partial features of ASIA. Given her typical ASIA symptoms - fever, fatigue, and sleep disturbance - and the improvement observed after removal of the suspected trigger, she met the diagnostic criteria and was diagnosed with ASIA.

## Discussion

This case illustrates a diagnostic challenge commonly faced by general physicians: persistent fever unresponsive to broad-spectrum antibiotics and unremarkable findings on standard imaging. In such scenarios, rare conditions such as ASIA should be considered, particularly in patients with a history of silicone breast implants, even when local breast symptoms are absent.

In this case, we systematically evaluated and ruled out potential causes of persistent fever, including infective endocarditis, autoimmune diseases, and malignancies. According to the diagnostic criteria for ASIA, a diagnosis requires the presence of either two major criteria or one major criterion accompanied by two minor criteria [1]. This patient met three major criteria: (1) exposure to an external stimulus (infection, vaccine, silicone, or adjuvant) before symptom onset; (2) presence of typical clinical manifestations such as fever, fatigue, and sleep disturbance; and (3) resolution of symptoms following removal of the offending agent. These findings strongly support the diagnosis of ASIA.

The patient also reported trauma preceding the onset of ASIA symptoms. Although existing literature has not established a direct relationship between trauma and ASIA development, and no causal link could be confirmed in this case, it is possible that the trauma contributed to implant rupture, thereby triggering immune activation.

Breast MRI played a key role in the diagnostic process by revealing abnormalities in the silicone implant. While MRI cannot confirm ASIA on its own, it is a highly accurate and reliable modality for assessing implant rupture and peri-implant inflammation [4,5]. Studies have shown that the diagnostic accuracy, sensitivity, and specificity of ultrasound in detecting breast implant rupture are 96%, 95%, and 96%, respectively, closely matching that of MRI [6]. Dual-energy CT has also shown comparable diagnostic performance [7]. However, MRI remains the preferred modality when ultrasound findings are inconclusive, and dual-energy CT is not widely accessible in routine practice. Thus, clinicians must recognize the utility of MRI in these clinical contexts.

In this case, despite the absence of localized breast findings, MRI successfully identified the ruptured implant, prompting surgical removal. This intervention marked a turning point in diagnosis and treatment, and the patient’s complete symptom resolution following explantation further supported the diagnosis of ASIA.

Clinicians should remain aware that silicone implants can be a hidden source of systemic symptoms such as fever, fatigue, and sleep disturbances, even in the absence of localized breast abnormalities. Early recognition of this rare condition, combined with appropriate use of breast MRI, may help prevent diagnostic delays and improve patient outcomes.

## Conclusions

This case highlights the importance of considering ASIA in patients with silicone breast implants who present with systemic inflammatory symptoms of unclear origin, even when local breast findings are lacking. Although MRI alone is insufficient to confirm the diagnosis, it plays a critical role in detecting implant rupture and peri-implant inflammation. Greater awareness of ASIA and proper imaging evaluation can facilitate timely diagnosis, reduce unnecessary treatments, and improve overall clinical outcomes.
